# The Legacies of Eugène Jamot and *La Jamotique*

**DOI:** 10.1371/journal.pntd.0002635

**Published:** 2014-04-24

**Authors:** Francois-Xavier Mbopi-Keou, Laurent Bélec, Jean-Marie Milleliri, Chong-Gee Teo

**Affiliations:** 1 Department of Laboratories and Blood Safety, Ministry of Public Health, and University of Yaounde I, Yaounde, Cameroon; 2 Laboratoire de Virologie, Hôpital Européen Georges Pompidou, and Faculté de Médecine Paris Descartes, Université Paris Descartes (Paris V), Paris, France; 3 UNAIDS, Dakar, Senegal; 4 Division of Viral Hepatitis, Centers for Disease Control and Prevention, Atlanta, Georgia, United States of America; Institute of Tropical Medicine, Belgium

*“On n'arrête pas un incendie avec un compte-gouttes.” (“You can't extinguish a fire with a dropper.”) —E. Jamot (attributed)* [1]

Who remembers “*l'éveilleur*” (“the awakener”), Colonel Eugène Jamot (1879–1937) [2]? Many still, it would seem, judging from the constant stream of biographies and articles that have appeared over the century, commemorating his contributions to the control of sleeping sickness, otherwise known as human African trypanosomiasis (HAT) [1], [3]. There is even an association that has been established and dedicated to his life and achievements [4].

## Jamot and *La Jamotique*

The young Jamot, who had earlier been stationed as a military physician in Algeria and Chad and undergone a stint in HAT research at the Pasteur Institute in Paris, had just been posted to the Institute at Brazzaville in French Congo (now Democratic Republic of the Congo) when he received mobilization orders to support action against the Germans in Cameroon. At the campaign's end and by the time of his return to Brazzaville, he had travelled across sufficiently vast stretches of French Equatorial Africa to have witnessed firsthand the devastation wrought by HAT. The Gambian form of this disease—caused by *Trypanosoma brucei gambiense*, transmitted by the tsetse fly, and increasingly rampant in the decades before the 20th century began—was rendering villages extinct [5].

**Figure 1:**
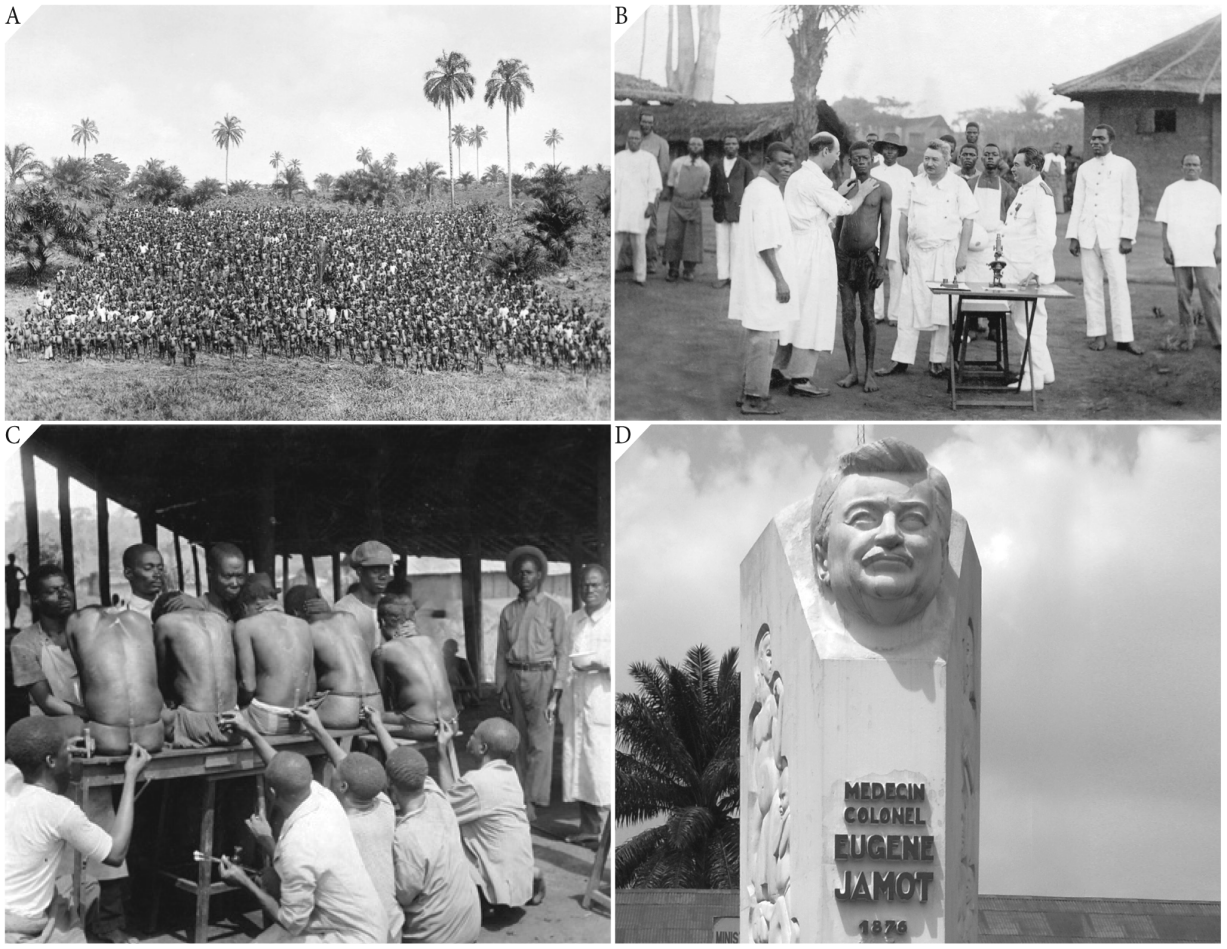
(A) Medical team with entire village, (B) open-air clinical examination (note Jamot in middle looking on and lone microscope on table in front), and (C) lumbar puncture conducted by medical auxiliaries; rural Cameroon. (D) Colonel Jamot's statue in front of the Department of Disease Control, Cameroon Ministry of Public Health, Yaounde, Cameroon.

Charged by the Governor General to lead a campaign against HAT, Jamot launched in 1916 a systematic and sustained offensive in Oubangui-Chari (now Central African Republic [CAR]). His approach to “medical prophylaxis” involved the dispatch on foot of survey teams, comprising medical auxiliaries and microscopists headed by a few physicians and nurses, followed up by independent treatment teams moving in to administer atoxyl to the HAT cases (Figure 1A–C). Within defined sectors, each village was visited and then revisited repeatedly to complete the course of trypanocidal injections. Jamot sought to eradicate HAT by extinguishing the trypanosomal reservoir rather than merely to impair the efficiency of vector transmission by suppressing the collective parasitemic load of the population. Within 18 months, nearly 90,000 people were examined, which led to >5,000 HAT cases being identified and receiving treatment [6].

In 1922, Jamot was assigned to Cameroon to set up an autonomous special service against HAT and was provided substantial resources from the Ministry of Colonies to prosecute the task. Having established headquarters and a training center in Ayos, he coordinated from there 28 mobile teams that included 17 physicians and 400 auxiliaries. Therapy now included use of tryparsarmide to improve the cure rate for the second (neuropsychiatric) stage of HAT. By 1930, up to 150,000 cases had been diagnosed and treated. HAT prevalence rates in the hyperendemic sectors dropped precipitously, e.g., from 60% to 4.1% in the Lomie subdivision and from 17.8% to <0.1% in the Yaoundé subdivision [7]. Jamot won wide acclaim for these accomplishments. His rapid-deployment, survey-and-treat approach to HAT eradication acquired the sobriquet *La Jamotique* [4].

## *L'affaire de Bafia*

The therapeutic ranges of atoxyl and tryparsamide (both arsenicals) were narrow because of toxicity targeted particularly at the optic nerve. When Jamot was on home leave in 1931, a junior colleague supervising the team then covering the region of Bafia increased the dosage of tryparsamide for treating advanced HAT. Some 700 cases allegedly became blind. Held responsible for this “therapeutic crime” [8], Jamot was dismissed from his post and assigned to Dakar, Senegal, and then to Ouagadougou in Upper Volta (now Burkina Faso). With a much smaller workforce, he still managed to treat 68,000 HAT cases in French West Africa. Continued opposition from detractors who criticized his achievements and the doctrine underlying them disheartened him. In failing health, Jamot retired in 1936, spending his last months working as a village doctor in Sardent, Creuse [1], [4].

The Bafia incident revived criticisms of Jamot's efforts to eliminate HAT. In French West Africa, *La Jamotique* was revoked in 1935, only to be restarted 4 years later. It was later replaced, in fits and starts, by organizational structures and systems directed at HAT (with vector control added), but over the decades these became polyvalent, orientated to tackle other major infectious diseases like yaws, syphilis, malaria, onchocerciasis, leprosy, and HIV/AIDS [5].

## Iatrogenic Transmission of Blood-Borne Pathogens

Infections with hepatitis C virus (HCV), hepatitis B virus (HBV), human T cell lymphotropic virus, and HIV-2, unlike HIV-1 infection, do not invariably or quickly kill human hosts. HCV infection is an especially good indicator of iatrogenic blood-borne infection because it is relatively widespread and transmits principally along parenteral and percutaneous routes. The extent to which iatrogenic transmissions contributed to HCV infection and by corollary to other blood-borne pathogens has been tracked using seroprevalence studies of antiHCV IgG and HCV viremia and molecular clock studies of HCV genetic sequences. In Cameroon, seroprevalence studies show up to 50% prevalence in people born before 1945, and molecular clock analyses of HCV reveal population expansions of genotypes 1 and 2 between 1920 and the 1940s and genotype 4 between 1910 and the 1940s. In the CAR, people who received trypanocidal treatment before 1951 have >3 times the risk of HCV infection compared to those who never received treatment. These various findings point to large-scale medical interventions conducted early in the 20th century in certain African territories as contributing to the endemicity of HCV and perhaps other blood-borne pathogens [9], [10].

To what extent might Jamot's mass survey-and-treat operations (Figure 1A–C) have led to the spread of blood-borne pathogens in sub-Saharan Africa? When he was implementing medical prophylaxis in Oubangui-Chari, atoxyl was administered by subcutaneous injection for HAT cases in the first (hemolymphatic) stage and injection into the spinal canal or intramuscularly for second-stage cases. That campaign used only six syringes [6]. Up until the end of January 1929, 135,185 atoxyl injections were administered in the Cameroon campaign [11]. Trypasarmide, when introduced, was also a parenteral drug, primarily for intravenous use.

Jamot was not unaware of safety concerns associated with injection of these trypanocidals. He applied stringent rules for disinfecting injecting equipment; e.g., both syringes and needles were to be boiled in water, and needles changed for each patient [11]. These rules, when followed, would have ensured disinfection but not sterilization. Given that *La Jamotique* was massive in scale and production line in nature, breakdowns and exigencies in the disinfection process were possible. Nonetheless, the frequency of such inadvertences and the extent to which they contributed to the endemicity of blood-borne infectious agents remain unknown, as many other mass interventions were being conducted during that period in history [9], [10].

## The Mobile Health Concept

American, Belgian, British, and French practitioners of mobile health credit Jamot as the founder of the concept [12], with one British veteran referring to it as “the characteristic French itinerant system” [13]. In modern-day Cameroon, the Ministry of Health has put concept to practice, having established the *Laboratoire National de Santé Hygiene Mobile* to diagnose HIV infection. Medical teams travel in customized vans to isolated villages, where rapid testing for HIV is conducted [14]. Testing will soon include HBV and HCV. Since its implementation in 2005, 186 villages have been reached (with 62 villages covered more than once), and 12 vans are now operational. By the end of 2012, 157,560 people were tested for HIV, and of these, 9,555 (6.1%) were found to be infected and referred to district medical centers for treatment. In rural Uganda, a variation of the mobile health concept involves community health workers who travel by motorcycle dispensing weekly antiretrovirals to HIV-infected patients in their homes [15].

## Conclusion

The name of Jamot continues to be revered, his achievements in controlling HAT epidemics not forgotten (Figure 1D). Jamot averred that for successful control of HAT it is necessary “to seek, to treat, and, if possible, to cure the sick” (“*doit rechercher les malades, les soigner et si possible les guérir*”) [8]. Setting up a healthcare facility (dispensary, clinic, or hospital) is inadequate to control, let alone eliminate, disease since, dependent as this approach is on people having the means to travel or to be brought to the facility, only passive detection of disease is enabled. Using specialized mobile teams for active case finding improves health care by allowing disease to be detected and then treated, and, if the disease is infectious, by blocking further transmission through removal or shrinkage of the pathogen reservoir. Systematic, active case detection of a disease or a group of diseases that may well be coexisting syndemically is the one lasting beneficent legacy of *La Jamotique*.
